# Chronic Vitamin C Deficiency Promotes Redox Imbalance in the Brain but Does Not Alter Sodium-Dependent Vitamin C Transporter 2 Expression

**DOI:** 10.3390/nu6051809

**Published:** 2014-04-29

**Authors:** Maya D. Paidi, Janne G. Schjoldager, Jens Lykkesfeldt, Pernille Tveden-Nyborg

**Affiliations:** Faculty of Health and Medical Sciences, University of Copenhagen, Frederiksberg C 1870, Denmark; E-Mails: maypa@sund.ku.dk (M.D.P.); jannes@sund.ku.dk (J.G.S.); jopl@sund.ku.dk (J.L.)

**Keywords:** vitamin C deficiency, SVCT2, redox imbalance, oxidative stress

## Abstract

Vitamin C (VitC) has several roles in the brain acting both as a specific and non-specific antioxidant. The brain upholds a very high VitC concentration and is able to preferentially retain VitC even during deficiency. The accumulation of brain VitC levels much higher than in blood is primarily achieved by the sodium dependent VitC transporter (SVCT2). This study investigated the effects of chronic pre-and postnatal VitC deficiency as well as the effects of postnatal VitC repletion, on brain SVCT2 expression and markers of oxidative stress in young guinea pigs. Biochemical analyses demonstrated significantly decreased total VitC and an increased percentage of dehydroascorbic acid, as well as increased lipid oxidation (malondialdehyde), in the brains of VitC deficient animals (*p* < 0.0001) compared to controls. VitC repleted animals were not significantly different from controls. No significant changes were detected in either gene or protein expression of SVCT2 between groups or brain regions. In conclusion, chronic pre-and postnatal VitC deficiency increased brain redox imbalance but did not increase SVCT2 expression. Our findings show potential implications for VitC deficiency induced negative effects of redox imbalance in the brain and provide novel insight to the regulation of VitC in the brain during deficiency.

## 1. Introduction

Vitamin C (VitC) has multiple roles in the brain acting both as a non-specific antioxidant [1,2] and as a co-factor of enzymatic reactions including collagen- and catecholamine synthesis [3,4], as well as being involved in neuronal glutamate re-uptake [3]. VitC is preferentially retained in the brain relative to other organs even during states of deficiency, emphasizing the prominence of this essential micronutrient in the brain [5,6]. Due to a high level of cellular metabolic activity and a high content of lipids, the brain, and particularly the growing brain, is prone to oxidative stress and lipid peroxidation [7]. Oxidative stress is defined as an imbalance in redox-homeostasis, e.g., between free radicals and the coherent protection by anti-oxidants [8]. Hence, oxidative stress is propagated when VitC supply is insufficient [9,10], thereby leading to imbalance in redox homeostasis and subsequent progressive cellular damage. Under such circumstances, lipid peroxidation has been a principal biomarker to assess oxidative damage in the brain and has been suggested to be associated with cognitive impairment and neurodegenerative diseases [11,12].

VitC enters the brain primarily in its reduced form, ascorbate (ASC) [13,14]. From the blood stream ASC is transported against a concentration gradient into brain through the choroid plexus to the cerebrospinal fluid (CSF) and from there reaches the brain to achieve homeostasis [3]. The majority of VitC transport to the brain is accomplished through an active transport by the sodium dependent vitamin C transporter (SVCT2), allowing VitC levels to reach concentrations 10 fold higher than that of the blood [15,16]. Mice lacking this transporter have been shown to die immediately after birth, displaying respiratory failure and hemorrhage in the brain [17,18]. A study with *Svct2*^+/−^ mice compared to wild type demonstrated that VitC levels in brain cortex were genotype dependent with higher VitC content in mice homozygous for *Svct2*^(+/+)^ compared to heterozygous counterparts [18], supporting the SVCT2 as the predominant active transporter to the brain. This is in agreement with findings of undetectable mRNA levels of *SVCT1* (which is present in other organs, e.g., the liver) in guinea pig brain homogenate [19] Within the brain differential VitC retention in specific brain regions has been reported [20]. Dietary intervention studies have shown increases in *Svct2* mRNA expression in the liver, both *in vitro* and *in vivo*, but not in the brain following VitC deficiency in mice unable to synthesize VitC (*Gulo*^−/−^) [21,22]. However, analysis of the *in vivo* SVCT2 expression in the cerebellum disclosed an increase in protein levels but not mRNA following VitC deficiency in adult *Gulo*^−/+^ mice. Although the increase was not significant, it showed an apparent response to reductions in VitC supply unlike in cortex of the same group, which was suggestive of tissue dependent regulation of SVCT2 [22,23].

Like humans and primates, guinea pigs cannot synthesize VitC due to mutation in the l-gulono-γ-lactone-oxidase gene and, therefore, VitC must be supplied in the diet to prevent the development of scurvy [24]. Hence, the guinea pig is considered a valuable model to investigate effects of VitC deficiency. We have previously shown that VitC deficiency in newly weaned guinea pigs elevates DNA repair and oxidative stress [25], and is associated with an impairment of spatial memory and reduction of hippocampal neurons in young guinea pigs with low levels of VitC [26]. In a recent study, we have shown a persistent hippocampal volume reduction in prenatally deficient animals regardless of postnatal VitC repletion [27].

In this study, we investigated if a chronic pre- and postnatal VitC deficiency in guinea pigs leads to postnatal (day 70, P70) lipid peroxidation in the brain, if prenatal damage persists following postnatal repletion, and if differences observed associate to gene and protein expression of SVCT2 transporter in three brain regions, hippocampus (HP), cerebellum (BC), and brain frontal cortex (BFC). We show that chronic pre- and postnatal VitC deficiency leads to redox imbalance by increase in ascorbate oxidation and malondialdehyde (MDA) in young guinea pig brains, however this does not result in changes in SVCT2 expression.

## 2. Experimental Section

### 2.1. Animal Experimentation

The study was approved by Danish Animal Experimentation Inspectorate (License No. #2007/561-1298) and in accordance with EU Directive 2010/63/EU for animal experiments. Animals were obtained as subsets in a large *in vivo* study [27]. Eighty pregnant Dunkin Hartley guinea pigs at gestation day 18 (Charles Rivers Lab, Kieslegg, Germany) were equipped with subcutaneous (s.c.) microchips for identification (PET-CHIP ID, e.vet^®^, Danworth farm, West Sussex, UK), and randomized into weight stratified dietary groups receiving sufficient (900 mg, *n* = 30) or deficient (100 mg, *n* = 50) levels of VitC per kg diet (quality controlled diets by Special Diets Services, SDS, Witham, UK). We have previously shown that the dose of 100 mg VitC/kg feed results in a non-scorbutic deficiency status in guinea pigs [26]. In this study, thirty female pups (*n* = 30) were included, forming three dietary groups differing only in VitC content of the feed: control (pre-/postnatally sufficient, CTRL, 900/750 mg vitC/kg diet), deficient (pre-/postnatally deficient, DEF, 100/100 mg vitC/kg diet) or repleted (prenatally deficient/postnatally sufficient, REPL, 100/750 mg vitC/kg diet). The animals were housed in floor pens and allowed feed, hay, and water *ad libitum*. They were weighed at least once a week. VitC status was verified by blood sampling (~300 µL) from *v. saphena* at its superficial course on tibia around postnatal day (P) 35 (data not shown).

### 2.2. Euthanasia

Animals were anesthetized by inhalation with isoflurane (Isoba Vet 100%, Intervet International, Boxmeer, The Netherlands). After disappearance of voluntary reflexes (palpebral and interdigital), thoracotomy was performed and an intracardial blood sample was obtained using a 5 mL syringe and 18G-needle previously flushed with 15% tripotassium-EDTA. Animals were sacrificed by exsanguination and subsequent decapitation. Blood samples were immediately centrifuged and stabilized. Brains were excised and weighed before sectioning through the cerebral longitudinal fissure. One hemisphere (randomized left/right) was subsequently intended for biochemical, gene and protein expression analysis; for gene and protein analysis HP, BC, and BFC were isolated and snap-frozen in liquid nitrogen. Remaining brain tissue was frozen on dry ice for biochemical analysis. All the excised tissues were stored at −80 °C until use. The paired hemisphere was stored for hippocampal volume assessment [27].

### 2.3. Biochemistry Analyses

Ascorbate and dehydroascorbic acid (DHA), the reduced and oxidized forms of VitC, respectively, in plasma and brain, as well as malondialdehyde (MDA) and glutathione in brain, were analyzed as described previously [28,29,30].

### 2.4. RNA Extraction and RT-PCR

RNA isolation was performed as described previously [19]. Briefly, approximately 25 mg of each of BC, BFC, and HP tissues were homogenized in trizol (InVitrogen, Merelbeke, Belgium) and precipitated with chloroform (Sigma, Steinheim, Germany) and isopropanol (Merck, Darmstadt, Germany). The resulting RNA was purified using spin columns according to manufacturer’s instructions (SV Total RNA Isolation System, Promega, Madison, WI, USA) and was eluted with 50 µL nuclease free water. The purity of RNA was determined by spectrometry (Nanodrop 2000; Thermo Scientific, Wilmington, DE, USA) with absorbance ratios A260/A280 and A260/A230. RT-PCR was performed with 2 µg of RNA in duplicates yielding a total volume of 50 µL cDNA for each sample (MmLV RT enzyme, 5× MmLV buffer and RNasin (Promega)); 10 mM dNTPs and Oligo (dT) primers (60 µg/120 µL) (Fermentas GmbH, St Leon Roth, Germany); Random hexamer primer (2 µg/µL) (GE Healthcare, Uppsala, Sweden).

### 2.5. Gene Expression Analysis

All cDNA samples were tested for DNA contamination with intron-spanning *beta-actin* primers (Table 1) prior to real time quantitative PCR (Q-PCR) and only included if negative for contamination. PCR products of included genes were confirmed by electrophoresis in 2% agarose gel, followed by PCR clean-up (PCR Clean Up System; Promega, Sweden) and subsequent sequencing of PCR products (LGC genomics, Berlin, Germany).

**Table 1:** Primers for PCR and quantitative PCR (Q-PCR).

Gene	Primer Sequence	Product size (bp)	NCBI Accession No.
*Beta-actin*	(F): GTAAGGACCTCTATGCCAACACA (R): ATGCCAATCTCATCTCGTTTTCT	346	[GenBank:AF508792]
*S18*	(F): ATGTGGTGTTGAGGAAAGCAG (R): GCTTGTTGTCCAGACCGTTG	195	[GenBank:XM_003473925.1]
*SVCT2*	(F): GTCCATCGGTGACTACTA (R): ATGCCATCAAGAACACAGGA	114	[GenBank:AF411585]

All primer sequences are presented in 5′–3′ direction (F): forward primer; (R): reverse primer. Sequence analysis confirmed the sequences similarity with the presented NCBI GenBank Accession numbers.

For Q-PCR analysis, efficiency generated from specific standard curves was applied to each run. Q-PCR was conducted (SYBR Green I master LC480 and LC480, Roche, Basel, Switzerland) in 96-well white plates (Roche, Mannheim, Germany) with triplicates of all samples (in dilution 1:5), nuclease free water as negative control and calibrator as positive control. Target gene expression analysis of *SVCT2* [31] from the three different brain samples was done by normalizing to the reference gene, *S18* (ribosomal protein S18). Primer sequences are displayed in Table 1.

### 2.6. Protein Extraction and Western Blot

Approximately 20 mg of brain tissue was homogenized in 250 µL ice cold radio-immuno-precipitation assay (RIPA) buffer with protease inhibitors (150mM sodium chloride, 1% Triton X-100, 0.5% sodium deoxycholate, 0.1% sodium dodecyl sulphate, 50 mM Tris, pH 8, 1:100 sigma complete protease inhibitor cocktail) and centrifuged at 12,000 rpm for 10 min at 4 °C. The resulting supernatant was transferred into aliquots and protein estimation was done using Bradford assay (Coomassie brilliant blue G-250 (Fluka, Damstadt, Germany), ethanol 96% (Danish distillers, Roskilde, Denmark), phosphoric acid 85% (Merck), 1 mg/mL bovine albumin (Sigma) with brain lysates diluted in PBS (Dulbecco’s, pH 7.4) in triplicates at 595 nm on a SpectraMax Plus 384 UV/VIS plate reader (Molecular Devices Inc., Sunnyvale, CA, USA).

Samples of approximately 30 µg of protein diluted with loading buffer and sample reducing agent (Invitrogen NuPAGE 4X LDS sample buffer, Invitrogen NuPAGE 10X sample reducing agent) were heated at 70 °C for 10 min and were loaded onto pre-cast polyacrylamide gels (Invitrogen NuPAGE 4%–12% Bis-Tris gels). All samples were run in duplicates and electrophoresis proteins were transferred to PVDF membranes (GE Health Care, Sarl Fribourg, Switzerland) in a semi wet blot chamber (TE 77PWR, Amersham Biosciences) at 169 mA for 45 min. Membranes were blocked for 1 h in 2% blocking buffer (Amersham ECL Prime Blocking Agent) diluted in wash buffer (1XPBS and 0.1% Tween) and incubated with primary antibody, SVCT2 (1:200, anti-goat IgG, Sc-9926, Santa Cruz Biotechnology, Dallas, TX, USA) or Actin (1:20000, Mouse Anti Actin IgG_1_, Millipore, Temecula, CA, USA) in blocking buffer at 4 °C overnight. Specificity of the antibody was tested by a pre-absorption test with blocking peptide for anti-SVCT2 (SC9926-P, Santa Cruz Biotechnology, Dallas, TX, USA). After washing the membrane, following secondary antibody incubation in 2% blocking buffer (1:4000, anti-goat IgG-HRP or 1:10,000, anti-mouse IgG-HRP, both from Santa Cruz Biotechnology) for one hour, the bands were visualized by enhanced chemiluminiscence (Amersham ECL Prime Western Blotting Reagent, UVP Biospectrum imaging system). Band location was identified by a Western protein standard (Magic Mark™ XP Western Protein Standard). Densitometry was performed with UVP Life Science Series Software. SVCT2 band intensities were initially normalized to respective actin bands. Normalization between blots was done to control sample that was also run in duplicates at the same position on all gels, as an internal control. SVCT2 expression was finally normalized relative to corresponding internal control.

### 2.7. Statistics

Differences in biochemistry, mRNA and protein expression were analyzed by using one-way ANOVA followed by Tukey’s multiple comparisons *post hoc* test in case of statistical significance. Variance homogeneity was analyzed by Levine’s test for equal variance and data transformation was done when Levine’s test for equal variance was significant (*p* < 0.05). All analyses were conducted using SAS/JMP version 8.0.

## 3. Results

### 3.1. Biochemical Analyses

As expected, VitC deficiency was reflected in the VitC concentrations in brain and plasma with lower levels in DEF animals and the %DHA in plasma and brain showing elevated oxidation of the VitC pool (*p* < 0.0001 for both; Table 2). No significant differences were found between VitC levels in CTRL and REPL groups demonstrating that REPL pups reached CTRL-status for VitC (Table 2). VitC status significantly affected brain MDA concentrations (*p* < 0.0001) with the DEF group displaying higher lipid oxidation compared to CTRL or REPL (*p* < 0.0001; Table 2). No significant difference in MDA level was found between CTRL and REPL groups at day 70. In contrast to MDA levels, dietary VitC did not affect brain glutathione levels significantly.

**Table 2:** Biochemical results from plasma and brain.

Biochemical results	CTRL	REPL	DEF	Effect
Plasma VitC (nmol/mL)	51.8 ± 22.4 ^a^	57.3 ± 13.6 ^a^	4.0 ± 3.2 ^b^	***
Plasma DHA% (% of total VitC)	10.2 ± 4.9 ^a^	7.7 ± 2.2 ^a^	19.1 ± 3.8 ^b^	***
Brain VitC (nmol/g tissue)	1399 ± 143 ^a^	1498 ± 42 ^a^	495 ± 252 ^b^	***
Brain DHA% (% of total VitC)	4.5 ± 2.8 ^a^	3.1 ± 2.2 ^a^	9.5 ± 4.6 ^b^	***
Brain MDA (nmol/g tissue)	313 ± 124 ^a^	258 ± 58.9 ^a^	476 ±106 ^b^	***
Brain GSH (nmol/g tissue)	1348 ± 112	1407 ± 44.6	1341 ± 101.0	-

Biochemistry results of CTRL, REPL, and DEF guinea pigs from brain and plasma following a prenatal VitC deficiency and subsequent two months postnatal repletion or deficiency, compared to pre-and postnatally sufficient controls. Effect of VitC diet between three groups by one way ANOVA marked *** *p* < 0.0001. All values are presented as means ± SD. Values with different superscript letters are significantly different (*i.e.*, “^a^” is different from “^b^”).

### 3.2. SVCT2 Expression

**Figure 1:**
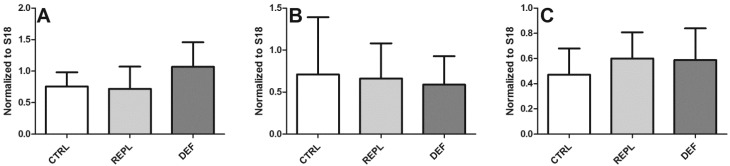
Quantitative PCR analysis of *SVCT2* mRNA expression in brain. Quantitative PCR analysis of *SVCT2* from three brain regions of guinea pigs between CTRL, REPL and DEF groups; Expressed values are mean of normalized ratio of *SVCT2* to the reference gene S18 ± SD; target gene expression in HP (**A**); BC (**B**); and BFC (**C**), *n* = 10 for each group. Effect of diet between the three groups was assessed by one way ANOVA (*p* > 0.05).

**Figure 2:**
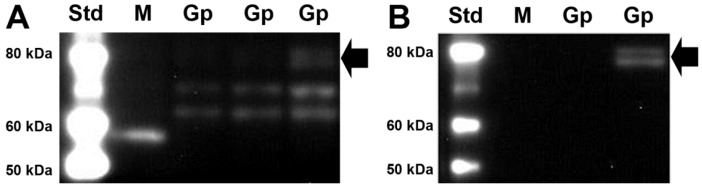
Specificity of anti-SVCT2 in Western blot. (**A**) Specificity of anti-SVCT2 without pre-absorption of antibody with blocking peptide in guinea pig (Gp) brain lysates (HP, BFC, and BC, respectively) seen as a doublet above 60 kDa and in mouse (M) brain lysates as a single band at 60 kDa; (**B**) Show brain lysates of Gp and M with pre-absorption of antibody with blocking peptide. Block arrow show the non-specific band detected in western blots in a few guinea pig brain lysates. Western blot standard ladder (Std) and band lengths are displayed for both blots.

**Figure 3:**
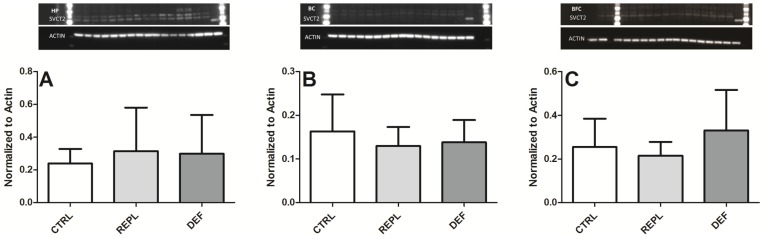
Western blot analysis of SVCT2 protein in brain. Densitometry analysis of western blot analysis of SVCT2 protein levels from three brain regions of guinea pigs HP (**A**); BC (**B**) and BFC (**C**); CTRL: control group, REPL: repleted group and DEF: deficient group. Representative pictures of the blots are depicted above each bar graph. Values are displayed as mean of normalized ratio to actin ± SD, the exact SVCT2 densitometry mean values (±SD) are provided in Table 3.

The PCR analysis of *SVCT2* expression from HP displayed a tendency to increase in DEF animals, but this was not significant (Figure 1A). No significant differences were observed in either BC or BFC regions between the three groups (Figure 1B,C). Western blot was performed to investigate if VitC deficiency affected SVCT2 protein levels from the three brain regions. Samples resulted in a doublet that may correspond to glycosylated and non-glycosylated forms (bands above 60 kDa and ~70 kDa) (Figure 2A), as has been previously reported [32,33]. Incubation with antibody specific blocking peptide successfully prevented both bands (Figure 2B), confirming specificity of the SVCT2 antibody in both guinea pig and mouse (positive control) lysates (Figure 2A,B). A few brain lysates gave rise to an additional 80 kDa band following anti-SVCT2 blots. This was detected even after pre-absorption with blocking peptide, confirming it to be a non-specific band (Figure 2B). Although we did not quantify band density between the three regions of the brain, hippocampus samples generally appeared to display intense bands compared to the samples from the other two brain regions with the faintest bands pertaining to samples from BFC. No significant changes in protein expression were found between the three dietary groups within the measured brain regions (Figure 3) suggesting that SVCT2 expression is not induced by the dietary regimes applied in this study.

**Table 3:** The exact SVCT2 densitometry mean values (±SD).

Densitometry	HP	BC	BFG
**CTRL**	0.2393 ± 0.0883	0.1632 ± 0.084	0.2549 ± 0.1294
**REPL**	0.3139 ± 0.2667 0.1299	0.1299 ± 0.043	0.2146 ± 0.063
**DEF**	0.2991 ± 0.2365	0.1380 ± 0.051	0.3305 ± 0.1858

## 4. Discussion

In the present study, we wanted to assess the effect of chronic pre- and postnatal VitC deficiency on oxidative stress markers and VitC transporter expression in the brain of young guinea pigs (P70), and if prenatal effects persisted after postnatal repletion. As expected, deficient animals had significantly lower levels of VitC in plasma while VitC levels did not differ between CTRL and REPL groups in spite of the difference in VitC status of their mothers (data not shown) [34]. DEF animals also showed increased oxidative stress measured by ascorbate oxidation (%DHA) and MDA. Other studies have reported MDA as a marker of lipid oxidation in the brain and increases in %DHA is associated to an enhance interaction between VitC and oxidants. The findings of both lipid oxidation and the promotion of VitC oxidation suggests increased levels of oxidative stress in DEF animals, which could result in disruption of the established metabolic pathways in the brain.

Low levels of VitC has been shown to increase GSH synthesis in brain of *Gulo*^−/−^ mice [35] and VitC has also been suggested to modulate GSH regulation in human erythrocytes [36]. Our data did not show any significant changes in brain total GSH suggesting that the chronically low levels of VitC in this study may still be sufficient to spare GSH in the brain. Moreover, VitC is capable of maintaining other anti-oxidants such as vitamin E in a reduced state [37]. However, previous investigations in VitC depleted guinea pigs have shown that despite the positive retainment of VitC in the brain during acute VitC deficiency, oxidative stress is not prevented and may have detrimental consequences for cellular function and survival [25].

No significant differences were found between CTRL or REPL groups for any of the measured parameters. The absence of a detectable difference between CTRL and REPL at P70 in both biochemical and molecular markers is likely due to VitC levels being restored at an earlier time point after repletion. We have recently reported that overall hippocampal volume was significantly reduced in prenatally deficient guinea pigs and persisted despite postnatal repletion [27]. This difference could be due to the hippocampal impairment occurring at a much earlier time-point (*i.e.*, prenatally), as opposed to the currently presented data which investigates animals at postnatal day 70, thus, much later than when the VitC deficiency was initially imposed. However, we have recently investigated the effects of prolonged maternal VitC deficiency in neonate (P7) guinea offspring [34]. Brain VitC levels in the neonate VitC deficient pups were 60% lower than the control group but there was no difference in ascorbate oxidation ratio between deficient and control pups. No effect of maternal VitC deficiency on either MDA or F_2_-isoprostanes in brain tissue of pups was found [34]. As the data is exclusively obtained in postnatal pups conclusions cannot be made concerning a potential VitC deficiency induced increase in fetal oxidative stress. However, the findings suggest that the increase in lipid oxidation and %DHA in the P70 guinea pigs included in the present study has occurred primarily as a result of postnatal deficiency.

In the brain, SVCT2 is the only known specific transporter that transports ASC into neurons [38,39]. Although mRNA expression of this transporter tended to increase in HP samples of the DEF group, the groups were not significantly different and this tendency was not confirmed by western blot. No significant differences in either gene or protein expression of SVCT2 was found in BFC or BC samples in coherence with reports from studies in mice [22,23]. Western blots with anti-SVCT2 showed double bands in all samples as has previously been reported [32,33]. Decreasing the total protein load [40], on the gels did not resolve the problem. Previous studies reported a range from 50 kDa to 65 kDa for SVCT2 in western blots from various tissues and species, and this is attributed to variable glycosylation and species specificity [40,41,42,43]. Although we did not find any significant changes in SVCT2 protein expression, we cannot rule out the possibility of specific regional increases within the investigated brain areas. It can also be speculated that SVCT2 levels may have increased during perinatal life only to have dropped by P70. Differences in SVCT2 expression during pre- and postnatal life has been shown in mice documenting a developmental regulation of this transporter [22,44]. However, no significant effect of postnatal VitC deficiency in *Gulo*^(−/−)^ mice on SVCT2 expression in the brain was reported although a tendency of an upregulation of SVCT2 protein in cerebellum of VitC deficient pups was proposed, indicating a regional-specific SVCT2 regulation [22].

Our results are in accordance with dietary intervention studies of VitC deficiency in *Gulo*^(−/−)^ mice, in which low levels of VitC resulted in increase in oxidative stress markers [20,35,45]. However, in these studies maternal environment was protected by supplementing the *Gulo*^(−/−)^ dams to meet the demands of pregnancy. VitC deficiency was thus imposed postnatally to the newborn pups. Maternal environment of the low VitC group in our present study is chronically low by gestational day 40 (data not shown) [34]. Thus, a consequence of a combined pre- and postnatal VitC deficiency is assessed as well as the effect of re-introducing high levels of VitC immediately after birth. We have recently shown that late-gestation guinea pig offspring from chronically VitC deficient dams display increased MDA and SOD in the brain indicating increased oxidative damage [46]. This supports that the prenatal development, e.g., the time of extensive brain growth, in guinea pigs is sensitive to VitC deficiency imposed effects. Whether the prenatal period is particularly vulnerable and if intervention with VitC repletion during this phase in development could prove to be protective of the reported hippocampal volume reduction [27] may be speculated. However, an exact “window of sensitivity” remains to be established, which may also include potential effects of prenatal *versus* postnatal life and hence also species differences, e.g., altricial *versus* precocial offspring.

VitC reaches the central nervous system through the choroid plexus, thus a differential regulation of the transporter particularly in this area remains a possibility. A study of regional brain ischemia in rats showed loss of *SVCT2* mRNA in the ischemic core followed by an increase in the peri-infarct regions at 22 h of reperfusion [47]. Similarly, the SVCT2 protein in brain was shown to increase on two days and five days following ischemia in mice and was also found to be increased around the ischemic core in areas where it would not usually be detected [48]. It should be noted, that the above studies have reported a difference in expression of this transporter two or five days after inducing ischemia, representing a short term effect as opposed to the chronic regime applied in our study.

Another possibility for not finding significant differences in brain SVCT2 expression corresponding to prolonged VitC deficiency would be because of tight regulation of the transporter and post-translational modifications. SVCT2 is supposedly glycosylated *in vivo* to maintain its functionality [39] and mutations in glycosylation sites of human SVCT2 significantly decreased VitC uptake in HepG2 cells [49]. Some *in vitro* studies have suggested that SVCT2 is regulated depending on the redox status of the cells showing an up-regulation of the transporter in the presence of oxidants [33,50]. Our findings propose that a chronic low VitC level is indeed associated with an increase in lipid peroxidation but does not increase SVCT2 in the measured brain regions. This could be due to variable stringency with which different factors can control the expression of SVCT2 in tissues.

## 5. Conclusions

In conclusion, chronic VitC deficiency during early life promoted postnatal redox imbalance in the brain, which was not observed in repleted animals. However, no association between dietary VitC and SVCT2 expression on either mRNA or protein level in the brain was observed. Our results suggest that modulation of SVCT2 expression within specific brain regions is not a potential mechanism to compensate for a chronic state of VitC deficiency in the brains of young guinea pigs.
